# Harnessing Tumor-Infiltrating Lymphocytes for Improved Cancer Outcomes

**DOI:** 10.30699/ijp.2025.2068603.3502

**Published:** 2025-11-11

**Authors:** Gunvanti Rathod, Pragnesh Parmar

**Affiliations:** 1 *Department of Pathology, AIIMS, Bibinagar, Telangana, India*; 2 *Department of FMT, AIIMS, Bibinagar, Telangana, India*

**Dear Editor,**

Cancer is not merely a disease of uncontrolled cell proliferation but also a complex interplay between malignant cells and the host immune system. Among the immune components infiltrating tumors, tumor-infiltrating lymphocytes (TILs) have gained prominence because of their dual role in tumor suppression and, paradoxically, tumor progression. TILs represent lymphoid cells that migrate from the peripheral blood into the tumor microenvironment (TME), and their composition, density, and spatial distribution are increasingly recognized as vital factors influencing tumor behavior and therapeutic response. The immune landscape of tumors, particularly the adaptive immune response mediated by TILs, reflects the dynamic interaction between cancer and the host. Advances in immunopathology and immuno-oncology have enabled researchers to characterize these lymphocytes and correlate their presence with clinical outcomes. The evaluation of TILs is now considered not only a valuable prognostic biomarker but also a guide for selecting patients likely to benefit from immunotherapies such as checkpoint inhibitors and adoptive T-cell therapies (1).

TILs are primarily composed of T lymphocytes, although B cells and natural killer (NK) cells may also be present. CD8⁺ cytotoxic T cells form the major effector arm and play a pivotal role in directly killing tumor cells by releasing perforin and granzymes. CD4⁺ helper T cells support cytotoxic T lymphocyte (CTL) activation and proliferation, whereas regulatory T cells (Tregs), often identified by FOXP3 expression, serve to suppress immune activation, thereby contributing to immune tolerance and sometimes facilitating tumor growth (2). In addition to cell type, the spatial distribution of TILs—whether within the tumor nests (intratumoral) or in the surrounding stroma (stromal)—can influence their functional activity and clinical relevance. The phenotypic characterization of TILs, including markers of activation (CD69, CD25), exhaustion (PD-1, TIM-3, LAG-3), and memory (CD45RO), provides insights into their functional status within the TME (3).

## Tumor-Immune Interaction and TIL Function

The tumor microenvironment is often immunosuppressive, favoring tumor escape mechanisms. Tumor cells can upregulate immune checkpoint molecules such as PD-L1 to engage PD-1 receptors on TILs, leading to T-cell exhaustion and impaired cytotoxicity. Infiltrating lymphocytes may also be exposed to immunosuppressive cytokines such as transforming growth factor β (TGF-β) and interleukin 10 (IL-10) secreted by tumor-associated macrophages and Tregs, further dampening the antitumor immune response (4). Despite these evasion strategies, TILs may exert significant antitumor effects. They recognize tumor antigens presented via major histocompatibility complex (MHC) molecules on cancer cells, and their accumulation within tumors often correlates with immune-mediated tumor control. High infiltration of CD8⁺ T cells, in particular, is associated with improved disease-free and overall survival in multiple carcinoma types (5).

TILs have demonstrated prognostic relevance in several cancers, especially breast, colorectal, lung, and ovarian carcinomas. In triple-negative breast cancer (TNBC) and human epidermal growth factor receptor 2 (HER2)-positive breast cancer, high stromal TILs are strongly associated with better response to neoadjuvant chemotherapy and improved survival outcomes. Salgado et al provided standardized guidelines for evaluating TILs in breast cancer, advocating quantification of stromal TILs as a percentage of stromal area (6). Their findings have been validated across multiple studies, showing that patients with high TILs have a significantly reduced risk of recurrence and death (7). In non–small cell lung carcinoma (NSCLC), the presence of CD8⁺ TILs has been linked with favorable prognosis. Studies have shown that high infiltration of these cells correlates with better survival outcomes and may predict responsiveness to checkpoint inhibitors such as nivolumab and pembrolizumab (8). Moreover, TILs expressing PD-1 in the presence of PD-L1 on tumor cells often signify an inflamed tumor microenvironment conducive to immunotherapy success (9). Colorectal carcinoma offers a unique model through the development of the “Immunoscore,” a standardized scoring system based on CD3⁺ and CD8⁺ TILs in the tumor center and invasive margin. Head and neck squamous cell carcinoma (HNSCC), particularly in human papillomavirus (HPV)-associated tumors, shows robust lymphocytic infiltration. These tumors generally have better outcomes, partly attributable to immune activation. High TIL levels in these cancers correlate with better locoregional control and overall survival (10).

## Predictive Value in Immunotherapy

One of the most transformative developments in oncology is the use of immune checkpoint inhibitors (ICIs). The presence of TILs in the TME, especially when combined with PD-L1 expression or a high tumor mutational burden (TMB), serves as a predictive biomarker for response to ICIs. TILs within an active immune contexture indicate a “hot” tumor that is more likely to respond to immune modulation. For instance, in melanoma and non–small cell lung carcinoma (NSCLC), patients with high TIL density often respond better to anti–PD-1 and anti–CTLA-4 therapies. The presence of TILs is also critical in determining eligibility for adoptive cell therapy (ACT), in which lymphocytes extracted from tumor tissue are expanded ex vivo and reinfused into the patient (10). The concept of TIL exhaustion is also relevant. TILs expressing multiple inhibitory receptors, while potentially dysfunctional, may still respond to checkpoint blockade therapy. Understanding and reversing this exhausted phenotype can enhance therapeutic efficacy.

**Table 1:** Summary of Key Studies on Tumor-Infiltrating Lymphocytes (TILs) in the Last 10 Years

Year	First Author	Cancer Type / Focus	Main Finding
2024	Elicora A6	Lung cancer (post-neoadjuvant chemotherapy)	Prognostic significance of CD4 and CD8 T-cell subgroups; higher CD8+ TILs linked to better outcomes
2024	Geurts VCM9	Triple-negative breast cancer (stage I, untreated)	High stromal TILs associated with favourable prognosis even without chemotherapy
2021	Blessin NC10	Multiple cancers	Proliferating CD8⁺ T cells are strong prognostic markers
2019	Loi S7	Early-stage triple-negative breast cancer	High TIL levels correlate with improved prognosis across pooled datasets
2018	Denkert C8	Breast cancer subtypes	Pooled analysis confirms prognostic value of TILs in TNBC and HER2+ cancers

According to Table 1, recent research has reinforced the prognostic and predictive significance of tumor-infiltrating lymphocytes (TILs) across various malignancies. Elicora et al (2024) demonstrated that in lung cancer patients receiving neoadjuvant chemotherapy, higher proportions of CD8⁺ T cells were significantly associated with improved survival, underscoring the role of cytotoxic lymphocytes in posttreatment tumor control (6). Geurts et al (2024) reported that in stage I triple-negative breast cancer (TNBC) patients who did not receive chemotherapy, elevated stromal TIL levels still predicted better prognosis, suggesting intrinsic antitumor immune activity independent of systemic therapy (9). Blessin et al (2021) found that proliferating CD8⁺ T cells served as a robust prognostic marker across multiple cancer types, indicating that both the quantity and proliferative capacity of TILs influence outcomes (10). Loi et al (2019), through pooled analysis of early-stage TNBC, confirmed that high TIL density strongly correlated with reduced recurrence risk and improved overall survival, supporting their inclusion in risk stratification models (7). Denkert et al (2018) extended these findings to HER2-positive breast cancer, showing that TILs were prognostic in multiple subtypes and predictive of chemotherapy benefit. Collectively, these studies highlight TILs—particularly activated and proliferating CD8⁺ subsets—as key biomarkers that reflect the tumor immune contexture, guide therapy selection, and may inform the design of immunotherapy strategies (8).

TILs are not only passive markers but are being actively harnessed for therapeutic purposes. Adoptive TIL therapy, which involves the expansion of TILs isolated from tumor tissue and reinfusion into the patient, has shown durable responses in melanoma and is being explored in cervical, ovarian, and non–small cell lung carcinoma (NSCLC) (13). Combining TIL therapy with checkpoint inhibitors, vaccines, or cytokines such as interleukin 2 (IL-2) or interleukin 15 (IL-15) may further enhance their antitumor activity. Advances in cellular engineering may enable modification of TILs to express chimeric antigen receptors (CARs), thereby increasing specificity and potency. Another novel approach involves enhancing TIL recruitment and retention at the tumor site using agents such as oncolytic viruses, which can both lyse tumor cells and stimulate immune infiltration.

Despite their potential, several challenges hinder the routine clinical use of TILs. First, there is variability in methods of TIL quantification across tumor types. Although the International TILs Working Group has established guidelines for breast cancer, similar consensus is lacking for other carcinomas. Furthermore, manual evaluation is subject to interobserver variability, although digital image analysis and machine learning tools are being developed to improve objectivity (6). Second, the heterogeneity of TILs in terms of phenotype and function complicates their interpretation. Not all TILs are beneficial; regulatory T cells (Tregs) and exhausted T cells may reflect immune suppression. Therefore, qualitative and functional assessments, such as cytokine production and checkpoint expression profiling, are needed alongside quantitative evaluations. Finally, TILs do not act in isolation. Their impact is influenced by other components of the tumor microenvironment (TME), including tumor-associated macrophages, myeloid-derived suppressor cells, and the microbiome. An integrated immunologic profile may be necessary to fully understand tumor-immune dynamics (10).

As a concluding remark, tumor-infiltrating lymphocytes are indispensable players in the tumor-immune interface. Their density, composition, and functional status have significant prognostic and predictive implications across various carcinomas. With the rise of immunotherapy and precision oncology, TILs are poised to become central not only as biomarkers but also as therapeutic agents. Standardizing their assessment and elucidating their functional diversity remain critical to fully exploiting their potential. As understanding of the immune contexture deepens, integrating TIL evaluation into routine clinical and pathological workflows will be pivotal for improving cancer outcomes.
